# Longitudinal study of gut microbiome in obsessive–compulsive disorder

**DOI:** 10.1002/brb3.3115

**Published:** 2023-06-05

**Authors:** Long Long Chen, Afrouz Abbaspour, Kristina Aspvall, Christian Rück, Cynthia M. Bulik, Diana Pascal

**Affiliations:** ^1^ Department of Clinical Neuroscience Centre for Psychiatry Research, Karolinska Institutet, & Stockholm Health Care Services Stockholm Sweden; ^2^ Department of Medical Epidemiology and Biostatistics Karolinska Institutet Stockholm Sweden; ^3^ Departments of Psychiatry and Nutrition University of North Carolina at Chapel Hill Chapel Hill North Carolina USA

**Keywords:** cognitive behavior therapy, diet, gastrointestinal microbiome, gut–brain axis, metagenomics, obsessive–compulsive disorder

## Abstract

**Introduction:**

Patients with obsessive–compulsive disorder (OCD) often have limited exposure to a diverse environment and perform repetitive compulsions such as excessive cleaning and washing, which could lead to altered gut microbiome. Therefore, longitudinal studies that investigate changes in gut microbiome before and after cognitive behavioral therapy based on exposure and response prevention (ERP) are warranted.

**Methods:**

All study participants (*N* = 64) underwent a structured psychiatric diagnostic interview prior to inclusion. Nutritional intake was assessed with a comprehensive food frequency questionnaire. Stool samples were collected from OCD patients before ERP (*n* = 32) and 1 month after completion of ERP (*n* = 15), as well as from healthy controls (HCs; *n* = 32). Taxonomic and functional analyses were performed using data from microbiome whole genome sequencing.

**Results:**

Patients with OCD at baseline reported consuming significantly less fiber than HCs (*R* ^2^ = .12, *F*(2, 59) = 5.2, *p* ≤ .01). There were no significant differences in α‐ and β‐diversity indices, or taxonomic dissimilarities at the species level between patients with OCD and HCs, or within patients before and after ERP. Functional profiling based on gut microbial gene expression was grouped into 56 gut–brain modules with neuroactive potential. None of the gut–brain modules differed significantly in expression between patients with OCD at baseline and HCs or within patients before and after ERP.

**Conclusions:**

The diversity, composition, and functional profile of the gut microbiome in patients with OCD did not differ significantly from HCs and remained stable over time, despite behavioral changes.

## Graphical abstract

The diversity, composition, and functional profile of the gut microbiome in patients with obsessive–compulsive disorder did not differ significantly from healthy controls and remained stable over time, despite behavioral changes after exposure and response prevention therapy.

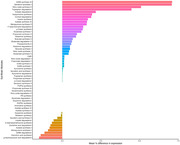

## 1 INTRODUCTION

Obsessive–compulsive disorder (OCD) is a debilitating neuropsychiatric disorder that affects 1.3% of the general population (Fawcett et al., 2020). OCD generally starts at an early age and usually takes a chronic course if left untreated (Stein, 2002). It is characterized by recurrent and persistent obsessions, which lead to repetitive and time‐consuming compulsions and/or avoidance. Patients with OCD are more confined to their home or certain environments, restricting their exposure to diverse surroundings (Brock & Hany, 2022). Certain compulsive behaviors, such as excessive cleaning and washing, further limit their exposure to the microbial environment we typically encounter every day. Other behaviors associated with OCD may also impact the diet, such as picky eating, and in extension the gut microbiome (Kauer et al., 2015).

The evidence‐based treatment for OCD is cognitive behavioral therapy with exposure and response prevention (ERP) (McKay et al., 2015). In this form of therapy, patients are encouraged to expose themselves to discomforting obsessions and learn to refrain from performing compulsions. ERP is an effective treatment for OCD and decreases the functional impairment of this disorder (Lundström et al., 2023). Whether behavioral changes achieved via ERP are associated with changes in the gut microbiome is a question that remains to be addressed.

The gut microbiome exerts extensive reciprocal interactions with our brain through microbial metabolites, the vagus nerve, and hormonal and immunological signaling, collectively forming the microbiome–gut–brain axis (Mayer, 2011). Systematic reviews on differences in gut microbiome between patients with psychiatric disorders and healthy controls (HCs) suggested that certain butyrate‐producing bacteria are less abundant among patients with psychiatric disorders (Chen et al., 2021; Nikolova et al., 2021). Specifically in patients with OCD, results from a pilot study showed that butyrate‐producing genera (*Oscillospira*, *Odoribacter*, and *Anaerostipes*) are lower in abundance compared with HCs (Turna et al., 2020). Butyrate, capable of regulating T‐cell generation, has important implications for our immune system (Arpaia et al., 2013). Pediatric acute‐onset neuropsychiatric disorder can be triggered by infections, such as group A streptococcus, leading to an auto‐immune response marked by obsessive–compulsive symptoms, which further strengthens the connection between bacterial pathogen, immunological response, and OCD (Gromark et al., 2019). However, many previous studies in the field are limited by study design and methodological shortcomings. There are ample opportunities to validate previous result with improved sequencing techniques and bioinformatic tools considering the recent advances in the field. As such, higher taxonomic resolution and coverage could be achieved using microbiome whole genome sequencing compared with 16S rRNA amplicon sequencing methods. Furthermore, whole genome sequencing provides comprehensive data on microbial gene content and allows for functional profiling.

The aim of this study was to compare the gut microbiome between patients with OCD and HCs, and changes in gut microbiome after ERP, using whole genome sequencing. Primary outcome measures are differences in taxonomic profiles and functional pathways.

## 2 MATERIALS AND METHODS

### 2.1 Study participants

Participants aged 18–45 years with a primary diagnosis of OCD according to DSM‐5 criteria were recruited from two organizationally and structurally equivalent publicly funded specialist OCD clinics in Stockholm, Sweden (Association, 2013). Sex‐ and age‐matched (±4 years) HCs with no personal history of OCD were recruited from the online platform Accindi.com. All participants underwent a combined structured clinical interview for DSM‐5 (SCID I) and Mini International Neuropsychological Interview (MINI) administered by an experienced specialist in psychiatry (American Psychiatric Association, 2013). Recruitment was conducted between November 2017 and February 2021. All study participants provided written informed consent. This study received ethical approval by Swedish Ethical Review Authority (D. nr. 2017/1711‐31/1) and was registered on ClinicalTrials.gov (NCT03638791).

A power calculation conducted on the HMP web application based on results from previous study by Turna et al. required a sample size of 30 participants in each group to reach a power estimate of 0.8 (File S1) (Mattiello et al., 2016; Turna et al., 2020).

### 2.2 Inclusion and exclusion criteria

Inclusion criteria for patients was a primary diagnosis of OCD. People from Stockholm County with no current psychiatric symptoms or diagnoses were recruited as HCs. Seven potential HC participants were excluded due to a current psychiatric diagnosis determined by the structured clinical interview.

Exclusion criteria for all participants were history of gastrointestinal tract surgery (other than appendectomy or cholecystectomy); history of inflammatory bowel disease, irritable bowel syndrome, celiac disease, or any other diagnosis that could explain chronic or recurring bowel symptoms; antibiotic use in the past 3 months; probiotic use in the past 4 weeks; pregnancy; intellectual disability; substance abuse; autism spectrum disorder; or psychotic disorders.

### 2.3 Exposure and response prevention therapy

Patients with OCD underwent exposure and response prevention (ERP) therapy, administered by experienced psychologists, either face‐to‐face, through videoconferencing, or internet based (Lundström et al., 2022). A minimum of five ERP sessions or five completed modules on the internet‐based psychotherapy were required for it to be considered a completed ERP treatment. Visits through videoconferencing and internet‐based therapy were accepted due to the restrictions of the COVID‐19 pandemic and contained the same therapeutic components as face‐to‐face ERP.

### 2.4 Data collection and clinical assessments

Study participants answered demographic questions through self‐report forms. Clinical assessment questionnaires were completed on the online platform BASS prior to sample collection, except for the Bristol stool scale, which was completed after stool sampling.

Obsessive–Compulsive Inventory—Revised (OCI‐R) is an 18‐item self‐report questionnaire estimating OCD symptom dimensions. The OCI‐R yields a total score and five subscales, including checking, hoarding, neutralizing, obsessing, ordering, and washing (Abramowitz & Deacon, 2006).

Eating Disorder Examination, revised (EDE‐Q) is a 28‐item questionnaire that assesses the range and severity of features associated with eating disorders (Fairburn & Beglin, 1994). This assessment was included to ensure that any differences in food intake were not due to the presence of an eating disorder in OCD patients, in whom eating disorder comorbidity is known to be high (Cederlöf et al., 2015).

MiniMeal Q questionnaire is a web‐based food frequency questionnaire based on normed data from Sweden that assesses habitual food habits. It includes 75–126 questions depending on the participant's declared food items that they consume (Christensen et al., 2014).

Bristol stool scale is a medical diagnostic scale designed to classify the form of human feces into seven categories.

The clinician‐rated Yale–Brown Obsessive–Compulsive Scale (Y‐BOCS), which is a semistructured interview assessing severity of OCD symptoms during the last week (Goodman et al., 1989), was administered by the same psychiatrist during the initial interview and at the follow‐up interview after ERP. Patients with at least a 35% decrease in Y‐BOCS score and posttreatment score on the Clinical Global Impression Scale—Improvement (CGI‐I) of 1 (“very much improved”) or 2 (“much improved”) were categorized as treatment responders (Mataix‐Cols et al., 2022).

### 2.5 Sample collection and sequencing

Participants were provided with clear instructions for stool sample collection at home using the OMNI‐Gut kit, which contains solution for DNA stabilization, making storage at room temperature possible without affecting sequencing. Patients with OCD were asked to collect an additional stool sample 1 month after the ERP treatment. The sampling kits were subsequently sent to the Karolinska Institutet Biobank and frozen at –80°C.

Sequencing was carried out in one single batch, and taxonomic profiling was finished on December 29, 2021 at Clinical Microbiomics, Denmark.

DNA was extracted from 250‐µL aliquots of the stool samples using the NucleoSpin^®^ 96 Stool (Macherey‐Nagel) kit. A minimum of one negative control and one positive control (ZymoBIOMICSTM Microbial Community Standard—Zymo Research) were included per batch of samples from DNA extraction and throughout the laboratory process.

The quality of the DNA samples was evaluated using agarose gel electrophoresis, and the quantity of the DNA was evaluated by Qubit 2.0 fluorometer quantitation. The genomic DNA was randomly sheared into fragments of around 350 bp. The fragmented DNA was used for library construction using the NEBNext Ultra Library Prep Kit for Illumina (New England Biolabs). The prepared DNA libraries were evaluated using Qubit 2.0 fluorometer quantitation. The library was sequenced using 2 × 150 bp paired‐end sequencing on an Illumina Novaseq 6000. A total of 79 stool samples were sequenced with average giga‐base‐base‐pair/sample of 7.6.

### 2.6 Bioinformatic analysis

Raw FASTQ files were filtered from human genome using reference genome GRCh38 on Bowtie2 (v. 2.4.2) (Langmead & Salzberg, 2012). Adapters were removed in addition to bases with a Phred score below 20 using AdapterRemoval (v. 2.3.1) (Schubert et al., 2016). A total of 96.8% of the base pairs had a Phred score above 20. Read pairs in which both reads passed filtering with a length of at least 100 bp were retained.

High‐quality nonhost reads were mapped to the Clinical Microbiomics in‐house Human Gut microbiome gene catalog (HG04) using BWA mem (v. 0.7.17) (Li & Durbin, 2009). For detailed description of the gene catalog, see File S2. The metagenomic species (MGS) concept has previously been described by Nielsen et al. (2014). An MGS is considered detected if genes from a sample uniquely mapped to at least three of its signature genes; measurements that did not satisfy this criterion were set to zero, resulting in 99.6% specificity.

Rarefied MGS abundance profiles were calculated by random sampling, without replacement, of a fixed number of signature gene counts per sample. In this study, 548,184 signature gene counts were sampled. The species tree for the MGS was created based on single‐copy bacterial and archaeal marker genes from the Genome Taxonomy Database (GTDB) (Chaumeil et al., 2019; Parks et al., 2018) (File S3). The species tree covered 2046 of 2095 MGSs and was rooted with archaea as an outgroup. Alpha‐ and β‐diversity estimates were calculated from rarefied abundance matrices.

EggNOG‐mapper (v. 2.1.7, Diamond mode) (Cantalapiedra et al., 2021) was used to map each gene in the gene catalog to the EggNOG (Huerta‐Cepas et al., 2019) orthologous groups database, resulting in EggNOG annotations for 81% of genes and Kyoto Encyclopedia of Genes and Genomes orthology database annotations for 39% of genes. INTERPROSCAN (v. 5.54‐87.0) was used to map the protein sequence encoded in each gene to the TIGFRAMs (v 15.0) database, resulting in TIGFRAM annotations for 21% of genes.

The gut–brain modules (GBMs) are a set of 56 microbial pathways for metabolizing neuroactive compounds (molecules that have the potential to interact with the human nervous system) (Valles‐Colomer et al., 2019). Based on the GBMs, gut microbial neuroactive metabolic profiles were generated by aggregating the abundance of all MGSs containing the given GBM.

### 2.7 Statistical analysis

All statistical analyses were performed in R version 4.0. There were generally few missing data, ranging from 3% to 5%. We used random forest imputation for relevant variables, otherwise shown as NA utilizing the R packages tidyverse and tidymodels (Kuhn & Wickham, 2020; Wickham et al., 2019). For demographic and clinical variables, comparison between patients with OCD and HCs was assessed using *t*‐tests for continuous variables and *χ* ^2^ tests for categorical variables (Pollard et al., 2018). Significance threshold was set to *p* < .05.

Nutrition data, the intake of food items, beverages, and dishes reported in the food frequency questionnaire Mini Meal Q (MMQ), were linked to the national database of nutrient contents published by the Swedish National Food Agency using an in‐house‐developed python script (Wiklund et al., 2022). We estimated differences in energy, macronutrient, and micronutrient intake between OCD and HCs, and within OCD cases before and after ERP using linear regression models adjusting for BMI. The Shapiro–Wilk test was used to assess the normality of the data and the assumptions for the regression models were adequately met.

Alpha‐diversity indices were estimated using nonparametric Wilcoxon rank sum test. Principal coordinate analysis (PCoA) was carried out using the ape package in R (Paradis & Schliep, 2019). Permutational analysis of variance (PERMANOVA) was performed on the distance matrices for β‐diversity indices, using the adonis function (Anderson, 2001). MGS counts at the species level were center‐log transformed and filtered down to features present in more than 25% of the participants. Dirichlet‐multinomial model was applied for differential abundance analysis using Welch's *t* and Wilcoxon rank test in ALDEx2 (Fernandes et al., 2013; Gloor et al., 2016). Linear regression models were applied on functional analysis of GBM. All statistical tests were conducted between cases (*n* = 32) and HCs (*n* = 32), and within cases (*n* = 15) before and after treatment separately. To correct for multiple comparisons, false discovery rate (FDR) based on Benjamini–Hochberg method was applied, and adjusted *p*‐values were set to a significance threshold of *q* < .05.

## 3 RESULTS

### 3.1 Demographics and clinical assessments

Thirty‐two patients with OCD and 32 sex‐ and age‐matched HCs were enrolled in this study. There were no statistically significant differences on any demographic variables (Table 1).

**TABLE 1:** Demographic characteristics of study participants.

	HC (*n* = 32)	OCD (*n* = 32)	*p*
Sex male (%)	13 (40.6)	13 (40.6)	1.000
Age (years), mean (SD)	28.62 (6.43)	27.60 (6.38)	.523
Weight (kg), mean (SD)	68.62 (13.65)	71.42 (16.35)	.461
Height (m), mean (SD)	1.71 (0.10)	1.73 (0.09)	.634
BMI, mean (SD)	23.32 (4.21)	23.84 (3.92)	.614
Country of birth (%)			.108
Sweden	24 (75.0)	27 (84.4)	
Abroad	8 (25.0)	3 (9.4)	
Highest education (%)			.299
Elementary school	11 (34.4)	15 (46.9)	
High school	20 (62.5)	14 (43.8)	
University degree	1 (3.1)	1 (3.1)	
Graduate degree	0 (0%)	0 (0%)	
Housing status (%)			.374
Lives alone	10 (31.2)	6 (18.8)	
Lives with children	16 (50.0)	18 (56.2)	
Lives with other adults	6 (18.8)	6 (18.8)	
Employment (%)			.333
Employed	27 (84.4)	23 (71.9)	
Unemployed	5 (15.6)	6 (18.8)	
Sick leave	0 (0.0)	1 (3.1)	

*Note*: *t*‐test for continuous variables and *χ* ^2^ test for categorical variables; significance threshold set to *p* < .05.

Abbreviation: BMI, body mass index.

Clinical characteristics are summarized in Table 2. All symptom dimensions except hoarding in OCI‐R were significantly higher among patients with OCD compared with HCs. Eating behavior assessed with EDE‐Q and stool sample classification with Bristol stool scale were not significantly different between cases and HCs. None of the participants had a score on EDE‐Q above the clinical cutoff for eating disorders (Fairburn & Beglin, 1994). Patients with OCD were more affected by psychiatric comorbidities, for example, major depressive disorder and anxiety disorder, as expected in a clinical population (Brakoulias et al., 2017). Moreover, 17 patients with OCD were stable on psychotropic medication at least 2 months before inclusion and during the ERP treatment, except for one patient who switched from dexamphetamine to atomoxetine.

**TABLE 2:** Clinical characteristics of study participants.

	HC (*n* = 32)	OCD (*n* = 32)	*p*
OCI‐R, mean (SD)			
Total score	5.91 (6.01)	25.48 (14.47)	<.001
Washing	0.78 (1.34)	3.92 (3.77)	<.001
Obsession	0.88 (1.43)	7.70 (3.70)	<.001
Hoarding	1.25 (1.72)	1.76 (2.89)	.392
Ordering	1.66 (1.86)	4.04 (3.80)	.002
Checking	0.97 (1.33)	5.24 (3.73)	<.001
Neutralizing	0.38 (0.98)	2.82 (4.02)	.001
Y‐BOCS, mean (SD)	NA	23.56 (5.35)	
EDE‐Q, mean (SD)	0.61 (0.66)	0.73 (0.73)	.503
Bristol‐scale, *N* (%)			.628
1	0 (0%)	0 (0%)	
2	2 (6.2)	2 (6.2)	
3	8 (25.0)	7 (21.9)	
4	16 (50.0)	16 (50.0)	
5	6 (18.8)	4 (12.5)	
6	0 (0.0)	1 (3.1)	
NA	0 (0.0)	2 (6.2)	
Comorbidity, *N* (%)			
Previous MDD	5 (16%)	7 (22%)	
MDD	0	6 (19%)	
Social phobia	0	4 (13%)	
GAD	0	4 (13%)	
Panic disorder	0	2 (6%)	
BDD	0	1 (3%)	
ADD	0	1 (3%)	
Dermatillomania	0	1 (3%)	

*Note*: t‐test for continuous data and *χ* ^2^ test for categorical data.

Abbreviations: ADD, attention deficit disorder; BDD, body dysmorphic disorder; EDE‐Q, Eating Disorder Examination Questionnaire; GAD, generalized anxiety disorder; MDD, major depressive disorder; MMQ, Mini Meal Q; OCI‐R, Obsessive–Compulsive Inventory—Revised; Y‐BOCS, Yale–Brown Obsessive–Compulsive Scale.

### 3.2 Exposure and response prevention

Among the participants with OCD at baseline, six did not complete their ERP treatment, and 11 patients were lost to follow‐up. Fifteen patients completed ERP treatment and provided stool samples 1 month after ERP. There was no significant difference in outcomes measured by Y‐BOCS score between participants who provided stool samples and those who were lost to follow‐up.

Fifteen patients with OCD completed ERP treatment, finished all assessments, and collected stool samples at 1 month posttreatment. Average length of ERP treatment was 3 months. Eight patients received ERP face‐to‐face and had at least nine sessions. Seven patients received internet‐based psychotherapy and finished at least nine modules. On average, the Y‐BOCS scores decreased by around 50% and OCI‐R score around 57% following ERP, whereas eating behavior assessed with EDE‐Q did not change (Table S1). Nine out of the 15 patients were responders, with a drop in Y‐BOCS score exceeding 35% and having a CGI‐I of 1 or 2.

### 3.3 Comparison of energy, macronutrient, and micronutrient intake

Mean intake of energy, macronutrients, and micronutrients for patients with OCD and HCs is summarized in Table 3. The two groups did not differ significantly on energy consumption and none of the participants reported energy intake below 800 kcal or above 5000 kcal. Similarly, patients with OCD had comparable intake of protein, fats, carbohydrates, and vitamins, but they consumed significantly less fiber (Christensen et al., 2014). The difference in fiber consumption remained significant after adjusting for BMI and correcting for multiple comparisons. Mean energy, macronutrient, and micronutrient intake did not differ significantly within patients with OCD before and after ERP treatment (Table S2).

**TABLE 3:** Nutrition report based on Food Frequency Questionnaire.

	HC (*n* = 32)	OCD (*n* = 30)	*q*
Energy (kcal), mean (SD)	2513.89 (1109.75)	2325.44 (866.62)	.51
Carbohydrate (g), mean (SD)	285.41 (136.14)	280.17 (106.99)	.77
Protein (g), mean (SD)	107.04 (57.87)	96.61 (41.27)	.51
Total fat (g), mean (SD)	96.36 (54.17)	84.38 (34.98)	.51
Saturated fat (g), mean (SD)	34.54 (22.39)	32.61 (14.71)	.65
Monounsaturated (g), mean (SD)	36.63 (21.47)	31.27 (13.43)	.49
Polyunsaturated (g), mean (SD)	15.92 (7.21)	12.76 (4.70)	.15
Fiber (g), mean (SD)	34.90 (17.32)	24.22 (9.23)	.04*
Vitamin C (mg), mean (SD)	107.71 (56.22)	76.13 (43.74)	.12
Vitamin D (µg), mean (SD)	5.54 (4.28)	4.86 (2.23)	.51
Vitamin B12 (mg), mean (SD)	4.94 (4.46)	4.42(2.5)	.61
Thiamin (mg), mean (SD)	1.67 (0.72)	1.42 (0.53)	.28

*Note*: Daily food intake per person based on Swedish nutrition data. Linear regression models adjusted for BMI. Significance level is set to FDR‐adjusted *p*‐value (*q* < 0.05).

### 3.4 Metagenomic analysis

Quality control of sequence reads and gene catalog mapping statistics for each sample can be found in File S3. On average, 86.3% of the high‐quality microbiome reads from a sample were mapped to the Clinical Microbiomics human gut gene catalog, and on average 297.2 MGSs were detected per sample.

#### 3.4.1 Diversity

Patients with OCD had lower species richness and phylogenetic diversity compared with HCs (Figure S1). However, none of the Wilcoxon test for α‐diversity indices, richness (*W* = 563, *p* = .76), Shannon index (*W* = 539, *p* = .64), or Faith's phylogenetic diversity (*W* = 550, *p* = .70), reached statistical significance. There were no significant changes in α‐diversity indices within the patients with OCD after ERP in terms of richness (*W* = 108, *p* = .43), Shannon index (*W* = 112, *p* = .5), or Faith's phylogenetic diversity (*W* = 106, *p* = .40).

**FIGURE 1:**
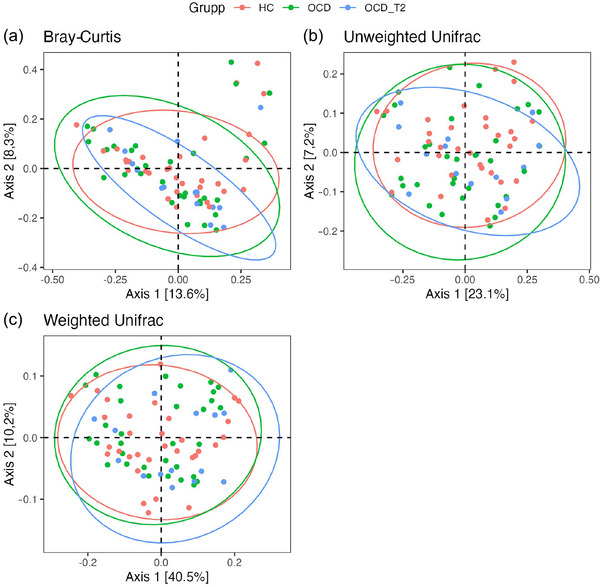
Principal coordinate analysis illustrating β‐diversity indices, (A) Bray–Curtis, (B) unweighted UniFrac distances, and (C) weighted UniFrac distances. Samples from HC are in red (*n* = 32), samples from patients with OCD are in green (*n* = 32), and samples from patients who have completed ERP treatment 1 month prior are in blue (*n* = 15). Multivariate analysis (PERMANOVA) for β‐diversity indices between the three groups were Bray–Curtis (*F* (2) = 0.02, *p*‐value = .97), unweighted UniFrac (*F* (2) = 0.02, *p*‐value = .96), and weighted UniFrac (*F* (2) = 0.02, *p*‐value = .50).

The gut microbiome composition of MGS (β‐diversity) largely overlaps between HCs, patients with OCD, and patients with OCD 1 month after ERP in the PCoA plots (Figure 1). Multivariate analysis (PERMANOVA) indicates that no significant differences in variance were detected using Bray–Curtis (*F* (2) = 0.02, *p* = .97), unweighted UniFrac (*F* (2) = 0.02, *p* = .96), and weighted UniFrac (*F* (2) = 0.02, *p* = .50) metrics between the three groups.

#### 3.4.2 Differential species abundance

**FIGURE 2:**
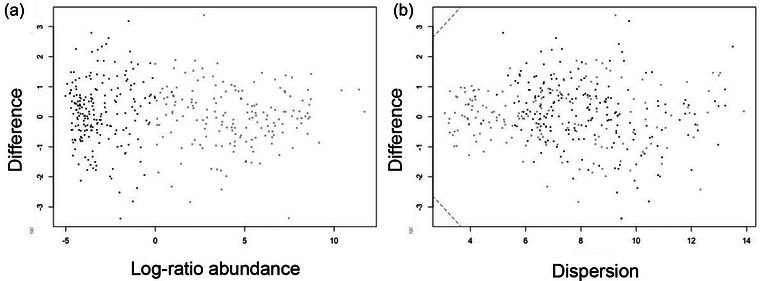
Differences in abundance at species level between patients with OCD and HC. (A) The left panel is a Bland–Altman plot that shows the relative abundance of species between OCD and HC. The log‐ratio abundance axis is the center‐log‐ratio value for the feature. (B) The right panel is an effect plot that shows the difference in effect size (dispersion) between OCD and HC. In both plots, each feature is represented with a dot (gray dots are abundant, while black dots are rare, neither are significantly differentially abundant).

There was no significant dissimilarity at the genus or species level between OCD and HCs based on FDR‐corrected *p*‐values and effect sizes (Figures 2 and S2). Similarly, no significant difference in abundance of genus or species was found within participants before and after ERP (Figures S3 and S4).

#### 3.4.3 Functional profiling with GBMs

**FIGURE 3:**
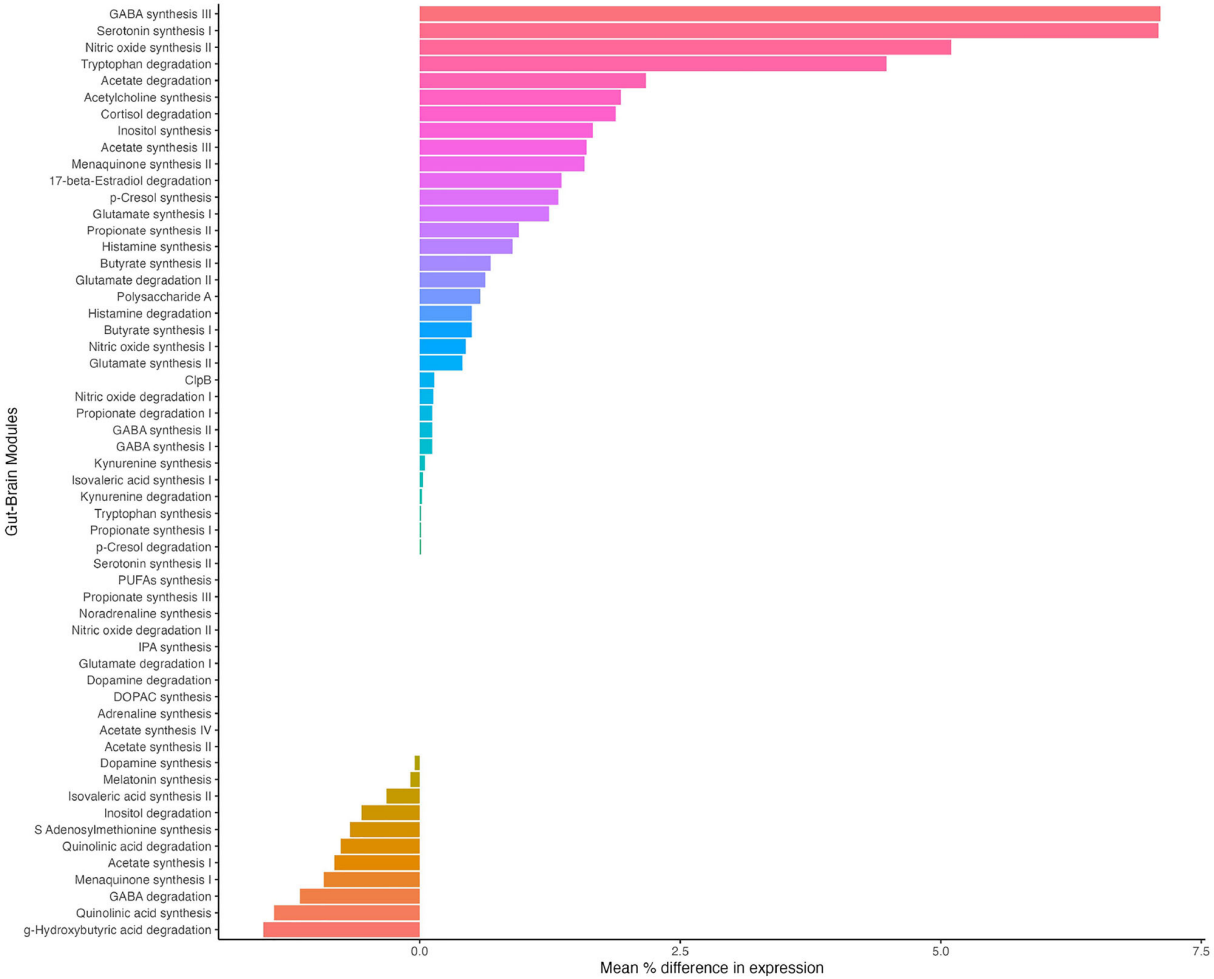
Differences in expression of neuroactive GBM between patients with OCD and HC as mean percentage. None of the GBMs reached statistical significance after correcting for multiple comparisons using FDR‐adjusted *p*‐value (*q* < .05).

Of the 56 GBMs described previously by Valles‐Colomer et al. (2019), 50 were detected at least once in all the samples and on average 41.7 GBMs were detected within each sample. The relative abundance of each GBM across all samples is shown in Figure S5. The estimated difference in expression of the GBMs between patients with OCD and HCs is shown in Figure 3. There were no statistically significant differences in GBMs either between HCs and patients with OCD or within patients with OCD before and after ERP, when FDR was applied (Figure S6; Tables S3 and S4).

## 4 DISCUSSION

This is the first longitudinal study on gut microbiome of patients with OCD before and after ERP treatment that is based on whole genome sequencing.

### 4.1 Nutrition

Data from food frequency questionnaires show that energy consumption is similar between patients with OCD and HCs. Serum levels of micronutrients, including B12, vitamin E, and vitamin C, have been found to be lower in patients with OCD (Balandeh et al., 2021). However, patients with OCD do not report consuming less vitamins based on the micronutrient intake from food frequency questionnaires in our study. Lower level of vitamin D could be explained by less exposure to sunshine (Marazziti et al., 2021).

In the study by Domènech et al. (2022), no dietary differences were found between OCD cases and HCs; however, a comprehensive food questionnaire linked to the national database of nutrient contents was not used. Fiber intake of patients with OCD in our study is below the recommended intake based on Nordic Nutrition Recommendations from 2012, but above the actual intake based on data from the Swedish Food Agency (Mithril et al., 2012; Saha et al., 2019). Time‐consuming obsessions and compulsions might affect choice and preferences for different food items. To avoid certain symptoms such as contamination or risk of harming others by using knives, patients with OCD could favor processed food products, which have lower fiber content. Some studies suggest that certain diets, especially a Mediterranean‐style diet rich in fiber, could reduce depressive symptoms and increase perceived mental health (Parletta et al., 2019). Moreover, dietary fiber intervention can increase concentration of short‐chain fatty acids (SCFAs), such as butyrate, in the gut (So et al., 2018). The gut microbiome is essential for the degradation of fiber into SCFAs, which has been linked to both anti‐inflammatory and neuroprotective effects (Alpino et al., 2022).

### 4.2 Gut microbiome

We observed no significant difference in α‐diversity metrics between patients with OCD and HCs, which aligns with results from two previous studies on gut microbiome in patients with OCD, except for the inverse Simpson index, which was lower in patients with OCD reported by Turna et al. (Domènech et al., 2022; Turna et al., 2020). The gut microbiome composition of HCs and patients with OCD both before and after ERP overlaps in the PCoA plots. No statistically significant differences were observed in microbiome composition indicated by β‐diversity measures among the three groups. This result is also consistent with the results from the two previous studies (Domènech et al., 2022; Turna et al., 2020).

There were no replicated findings at the genus level between our study and the two previous studies in patients with OCD (Domènech et al., 2022; Turna et al., 2020). Some findings are even contradictory—Turna et al. reported lower abundance of butyrate‐producing genera *Oscillibacter* and *Anaerostipes*, whereas Domènech et al. found higher abundance of those genera among patients with OCD. Methodological differences could partly explain the inconsistency in the results (Chen et al., 2021). We used whole genome sequencing rather than the 16S method, and dissimilarity analysis was performed at the species level compared with genus level in the two other studies, thereby providing a more detailed comparison of differences in gut microbiome.

Nevertheless, singular taxonomic dissimilarities in abundance cannot fully explain differences in functionality in the gut microbiome (Zhang et al., 2019). There is also a redundancy in gene expression meaning that the same genes are present in different taxonomic species. Therefore, functional analysis of the gut microbiome is essential for elucidating the relationship between gut microbiome and brain functions. By selecting a group of relevant functional pathways, we could compare the neuroactive potential of the gut microbiome (Valles‐Colomer et al., 2019). However, no significant difference in any of the GBMs between patients with OCD and HCs was observed. The largest increase in expression was GABA synthesis and serotonin synthesis, whereas the largest decrease in expression was degradation of hydroxybutyric acid. Studies using mainly 16S methods have found lower abundance of butyrate‐producing genera in psychiatric disorders; however, studies using adequate functional analysis are warranted to further investigate the relevance of those results (Chen et al., 2021).

Longitudinal studies that investigate temporal differences in gut microbiome in psychiatric disorders are limited (Chen et al., 2021). Neither the diversity nor composition of the gut microbiome changed significantly after ERP treatment in our study. The only other study with longitudinal data (3 months after initial sampling) did not show any difference either, although a trend toward higher α‐diversity was stated at the second time point (Domènech et al., 2022). The stability in gut microbiome composition in the present study is further supported by the lack of significant differences in abundance before and after ERP at the species level. Regarding functional analysis of neuroactive GBM, the difference in expression was limited to less than 3% and none of the results reached statistical significance. These findings support a stable gut microbiome at the group level over time in spite of behavioral changes (Faith et al., 2013).

First, a limitation to this study is the small sample size. Second, since the recruitment of cases was in a clinical setting, most patients with OCD were on medication. Some studies suggest that selective serotonin reuptake inhibitors (SSRIs) could affect the gut microbiome, but controlling for medication did not affect the results in a similar study to ours (Weersma et al., 2020). Lastly, only half of the participants completed the ERP treatment and collected stool sample 1 month later, which limits the conclusion we can draw from the longitudinal data.

## 5 CONCLUSION

This study does not concur with results from previous studies reporting altered gut microbiome in patients with OCD. In addition, reduction of OCD symptoms after ERP did not change the composition or functionality of the gut microbiome during the studied time course of 4 months, which further weakens the proposed association. Larger studies are necessary to determine whether the gut microbiome in individuals with OCD differs from HCs prior to recommending therapeutic interventions aimed at changing the gut microbiome in patients with OCD. It could be valuable to focus on patients with specific compulsions, such as washing and cleaning, to further evaluate the impact of behavior on gut microbiome.

## AUTHOR CONTRIBUTIONS

Diana Pascal, Cynthia M. Bulik, and Long Long Chen conceptualized the project. Long Long Chen and Kristina Aspvall set up the infrastructure for this project. Long Long Chen and Diana Pascal recruited participants for this project. Long Long Chen and Afrouz Abbaspour performed the analysis. All authors contributed to the writing and revising of the manuscript.

## CONFLICT OF INTEREST STATEMENT

C. M. Bulik: Shire (grant recipient, Scientific Advisory Board member); Lundbeckfonden (grant recipient); Pearson (author, royalty recipient); and Equip Health Inc. (Stakeholder Advisory Board). The other authors declare no conflicts of interest.

### PEER REVIEW

The peer review history for this article is available at https://publons.com/publon/10.1002/brb3.3115

## Supporting information

Supplementary file 1

Supplementary file 2

Supplementary file 3

Supplementary Table 1. Differences in symptoms before and after ERP (n = 15)
Supplementary Table 2. Differences in nutrition based on FFQ before and after CBT
Supplementary Figure 1. Comparison of alpha diversity indices (richness, Shannon index, Faith's phylogenetic diversity) between healthy controls and patients with OCD (A, B, C), and between patients with OCD before and after CBT treatment (D, E, F).
Supplementary Figure 2. Differences in abundance at the genus level between patients with OCD and healthy controls (HC).
Supplementary Figure 3. Differences in abundance at the species level within patients with OCD before and after ERP.
Supplementary Figure 4. Differences in abundance at the genus level within patients with OCD before and after ERP.
Supplementary Figure 5. Overview of neuroactive potential profiles.
Supplementary Table 4. Differences in expression of GBM between patients with OCD and healthy controls
Supplementary Figure 6. Differences in expression of neuroactive gut‐brain module (GBM) between patients with OCD at baseline compared with same patients one month after ERP treatment depicted as mean percentage. None of the GBMs reached statistical significance after correcting for multiple comparisons using FDR adjusted p‐value (q < 0.05).
Supplementary Table 5. Differences in expression of GBM within patients with OCD before and after CBT

## Data Availability

Metagenomic raw data were deposited in the Sequence Read Archive (SRA) under the accession number PRJNA883179.
