# Supplementary material for: Longitudinal study of gut microbiome in obsessive–compulsive disorder
Source: Brain Behav. 2023 Jun 5;13(8):e3115. doi: 10.1002/brb3.3115 (PMC10454283; doi:10.1002/brb3.3115)
Supplement: Supplementary file 1 [file BRB3-13-e3115-s002.pdf]

Sample Size Determination

HMP web application was used for determining power and sample size based on results from a previous study by Tuna et al. (10, 14). A sample size of 30 participants in each group reached a power estimate of 0.8.

INPUTS

Description	Value	Description	Value
Stratification	YES	Sample 1 Size	22
MonteCarlo Repl.s	100	Sample 2 Size	21
Pi-One type	StoolEntero	Min Sample Size	5
Number of OTUs	3	Max Sample Size	70
Changed OTUs 1	1	Rel. Abund. Diff. 1	50 %
Changed OTUs 2	2	Rel. Abund. Diff. 2	200 %
Most/Least Abundant 1	most abundant	Significance Level	0.05
Most/Least Abundant 2	most abundant		

Theta parameter instead is.

```
## Bacteroides  Prevotella Ruminococcus
##      0.052      0.054      0.051
```

OUTPUTS

Settings graphs

The following graphs show desired relative abundance distributions with their respective estimates based on the two simulated samples of size 22 and 21, respectively.

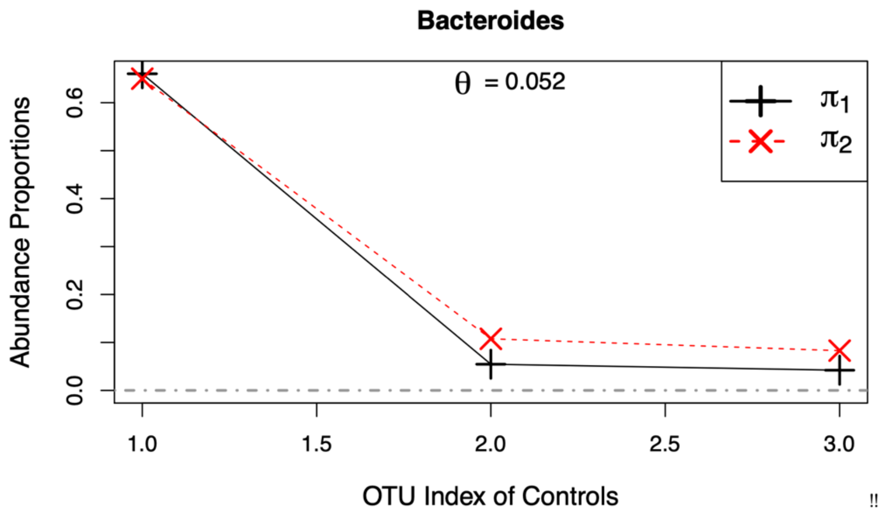

!!

Library Size Distribution

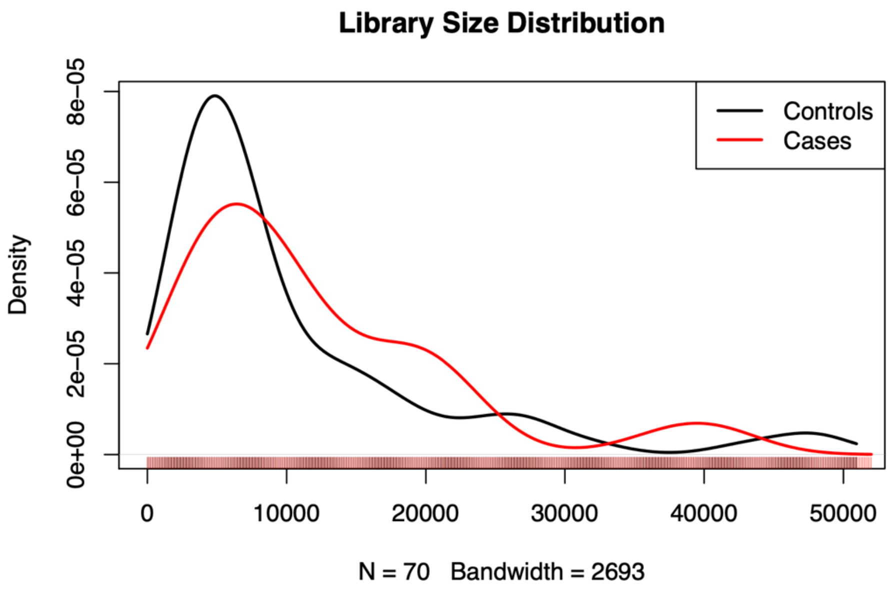

Power simulation with current settings

Power results for the current settings:

Wald WMW WMW avg
Bacteroides 0.50 0.58 0.253
Prevotella 0.68 0.51
Ruminococcus 0.59 0.86
Global 0.89 1.00
0.230
0.460
0.623

Power and sample size graph
The next graph shows the **power** *vs.* the **sample size** behaviour where the latter goes from 5 to 70.

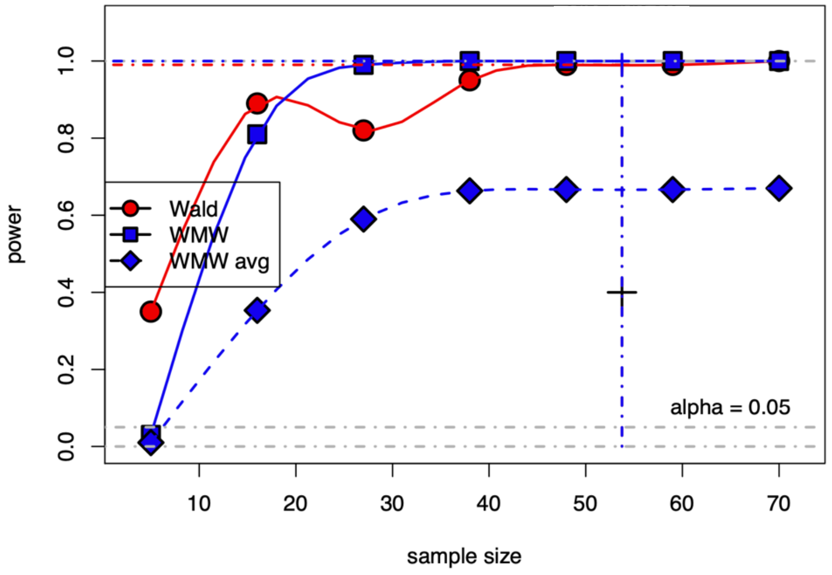

Power of individual OTUs

The following table appears only in case WMW test was selected. Column **Type** indicates the type of the OTU:

- type2DA: second type of Differentially Abundant OTUs, the ones with the bigger default effect size (percentage increase);
- type1DA: first type of Differentially Abundant OTUs, the ones with the smaller default effect size.

Bacteroides.ID	Bacteroides.Power	Type
Bacteria.Bacteroidetes.Bacteroidia.Bacteroidales.Bacteroidaceae.Bacteroides	0.660	type1DA
Bacteria.Bacteroidetes.Bacteroidia.Bacteroidales.Porphyromonadaceae.Parabacteroides	0.055	0
Bacteria.Bacteroidetes.Bacteroidia.Bacteroidales.Rikenellaceae.Alistipes	0.042	0

Prevotella.ID	Prevotella.Power	Type
Bacteria.Bacteroidetes.Bacteroidia.Bacteroidales.Prevotellaceae.Prevotella	0.257	type1DA
Bacteria.Bacteroidetes.Bacteroidia.Bacteroidales.Bacteroidaceae.Bacteroides	0.224	0
Bacteria.Bacteroidetes.Bacteroidia.Bacteroidales.Rikenellaceae.Alistipes	0.053	0

Ruminococcus.ID	Ruminococcus.Power	Type
Bacteria.Bacteroidetes.Bacteroidia.Bacteroidales.Bacteroidaceae.Bacteroides	0.307	type1DA
Bacteria.Firmicutes.Clostridia.Clostridiales.Ruminococcaceae.Faecalibacterium	0.084	0
Bacteria.Firmicutes.Clostridia.Clostridiales.Ruminococcaceae.unclassified	0.074	0
