# Supplementary material for: Longitudinal study of gut microbiome in obsessive–compulsive disorder
Source: Brain Behav. 2023 Jun 5;13(8):e3115. doi: 10.1002/brb3.3115 (PMC10454283; doi:10.1002/brb3.3115)
Supplement: Supplementary file 2 [file BRB3-13-e3115-s001.docx]

Supplementary file 2

**Clinical Microbiomics Human Gut HG04 gene catalog**

As a reference gene catalog, we used the Clinical Microbiomics in-house Human Gut HG04 gene catalog (14,355,839 genes), which was created based on 12,170 non-public deep-sequenced human gut specimens (including 481 from infants), 9,428 publicly available metagenomes compiled from 43 countries (1) and 3,567 publicly available genome assemblies from isolated microbial strains. For taxonomic abundance profiling, we used the Clinical Microbiomics in-house HGMGS version HG4.D.2 set of 2,095 metagenomic species (MGS), each represented by a set of genes with highly coherent abundance profiles and base compositions in the 12,170 metagenomes. The metagenomic species concept is described in Nielsen et al. 2014, (2).

**MGS taxonomical annotation**

To taxonomically annotate an MGS, we blasted its genes against NCBI RefSeq prokaryotic genomes (2022-01-19) and nt (2021-08-03) databases and used rank-specific annotation criteria. Specifically, we assigned a taxon to an MGS if at least M % of its genes were mapped to the taxon and no more than D % of its genes were mapped to a different taxon. We only considered blast hits with an alignment length ≥ 100 bp, ≥ 50 % query coverage and % identity ≥ PID. Here we define: PID = (95, 95, 85, 75, 65, 55, 50, 45); M = (75, 75, 60, 50, 40, 30, 25, 20); and D = (10, 10, 10, 20, 20, 20, 20, 15) for subspecies, species, genus, family, order, class, phylum, and superkingdom, respectively. Finally, we processed each MGS with CheckM (3), and updated our annotation with the CheckM result if this resulted in a lower taxonomic rank.

**Derivation of MGS-based species tree**

The species tree for the MGS was created based on single-copy bacterial and archaeal marker genes from the Genome Taxonomy Database (GTDB) consisting of 120 bacterial and 122 archaeal marker-genes belonging to either TIGRFAM or PFAM protein families. First, INTERPROSCAN (4) was used to identify marker genes within each MGS. Multi-copy marker genes and marker genes that were identified in < 10 MGS were excluded, resulting in a total of 111 bacterial and 26 archaeal marker genes with sufficient coverage. 7 of the 130 marker genes were shared between bacteria and archaea. MGSs with fewer than 10 marker genes identified by this method and MGS that were annotated as eukaryotes were excluded. Protein sequences from these 130 marker genes were aligned using HMMalign (v.3.2.1) and non-aligned residues were trimmed from the multiple sequence alignment. The species tree was next inferred using the concatenation-based species tree approach in IQtree (v.2.1.2) (5) with 1000 ultrafast bootstraps (6) and an edge-linked partition model (7).

1. Pasolli E, Asnicar F, Manara S, Zolfo M, Karcher N, Armanini F, et al. Extensive Unexplored Human Microbiome Diversity Revealed by Over 150,000 Genomes from Metagenomes Spanning Age, Geography, and Lifestyle. Cell. 2019;176(3):649-62.e20.

2. Nielsen HB, Almeida M, Juncker AS, Rasmussen S, Li J, Sunagawa S, et al. Identification and assembly of genomes and genetic elements in complex metagenomic samples without using reference genomes. Nat Biotechnol. 2014;32(8):822-8.

3. Parks DH, Imelfort M, Skennerton CT, Hugenholtz P, Tyson GW. CheckM: assessing the quality of microbial genomes recovered from isolates, single cells, and metagenomes. Genome Res. 2015;25(7):1043-55.

4. Jones P, Binns D, Chang HY, Fraser M, Li W, McAnulla C, et al. InterProScan 5: genome-scale protein function classification. Bioinformatics. 2014;30(9):1236-40.

5. Nguyen LT, Schmidt HA, von Haeseler A, Minh BQ. IQ-TREE: a fast and effective stochastic algorithm for estimating maximum-likelihood phylogenies. Mol Biol Evol. 2015;32(1):268-74.

6. Hoang DT, Chernomor O, von Haeseler A, Minh BQ, Vinh LS. UFBoot2: Improving the Ultrafast Bootstrap Approximation. Mol Biol Evol. 2018;35(2):518-22.

7. Chernomor O, von Haeseler A, Minh BQ. Terrace Aware Data Structure for Phylogenomic Inference from Supermatrices. Syst Biol. 2016;65(6):997-1008.
