# Supplementary material for: Longitudinal study of gut microbiome in obsessive–compulsive disorder
Source: Brain Behav. 2023 Jun 5;13(8):e3115. doi: 10.1002/brb3.3115 (PMC10454283; doi:10.1002/brb3.3115)
Supplement: Supplementary file 4 — Supplementary Table 1. Differences in symptoms before and after ERP (n = 15) Supplementary Table 2. Differences in nutrition based on FFQ before and after CBT Supplementary Figure 1. Comparison of alpha diversity indices (richness, Shannon index, Faith's phylogenetic diversity) between healthy controls and patients with OCD (A, B, C), and between patients with OCD before and after CBT treatment (D, E, F). Supplementary Figure 2. Differences in abundance at the genus level between patients with OCD and healthy controls (HC). Supplementary Figure 3. Differences in abundance at the species level within patients with OCD before and after ERP. Supplementary Figure 4. Differences in abundance at the genus level within patients with OCD before and after ERP. Supplementary Figure 5. Overview of neuroactive potential profiles. Supplementary Table 4. Differences in expression of GBM between patients with OCD and healthy controls Supplementary Figure 6. Differences in expression of neuroactive gut‐brain module (GBM) between patients with OCD at baseline compared with same patients one month after ERP treatment depicted as mean percentage. None of the GBMs reached statistical significance after correcting for multiple comparisons using FDR adjusted p‐value (q < 0.05). Supplementary Table 5. Differences in expression of GBM within patients with OCD before and after CBT [file BRB3-13-e3115-s003.docx]

| Supplementary Table 1. Differences in symptoms before and after ERP (n=15) | | | |
| --- | --- | --- | --- |
|  | Pre-treatment | Post-treatment | p-value |
| Y-BOCS | 24.71 (4.89) | 12.29 (6.80) | <0.01 |
| OCI-R | 27.93 (15.13) | 12.07 (7.76) | <0.01 |
| EDE-Q | 0.55 (0.65) | 0.57 (0.77) | 0.88 |
| Paired *t*-test was applied, significance threshold set to p<0.05. OCI-R (Obsessive Compulsive Inventory Revised), YBOCS (Yale-Brown Obsessive-Compulsive Scale), EDE-Q (Eating Disorder Examination Questionnaire). | | | |

| Supplementary Table 2. Differences in nutrition based on FFQ before and after CBT | | | |
| --- | --- | --- | --- |
|  | OCD before ERP (n=15) | OCD after ERP (n=15) | q |
| Energy (kcal) (mean (SD)) | 2348.0 (1057.7) | 2433.3 (804.9) | 0.81 |
| Energy (kj) (mean (SD)) | 9821.3 (4424.7) | 10178.5 (3366.1) | 0.81 |
| Carbohydrate (g) (mean (SD)) | 281.0 (126.1) | 285.42 (110.1) | 0.92 |
| Protein (g) (mean (SD)) | 96.9 (50.9) | 100.9 (40.9) | 0.82 |
| Total fat (g) (mean (SD)) | 86.0 (41.3) | 91.2 (30.6) | 0.71 |
| Saturated fat (g) (mean (SD)) | 33.1 (16.7) | 34.9 (12.7) | 0.75 |
| Monounsaturated (g) (mean (SD)) | 31.6 (15.9) | 33.3 (11.9) | 0.76 |
| Polyunsaturated (g) (mean (SD)) | 13.3 (5.9) | 14.2 (5.2) | 0.70 |
| Fiber (g) (mean (SD)) | 25.8 (9.5) | 28.9 (10.5) | 0.42 |
| Vitamin C mg (mean (SD)) | 80.59 (52.12) | 76.83 (42.51) | 0.836 |
| Vitamin D µg (mean (SD)) | 4.91 (2.41) | 5.40 (2.44) | 0.601 |
| Vitamin B12 mg (mean (SD)) | 4.60 (2.70) | 5.24 (2.48) | 0.524 |
| Thiamin mg (mean (SD)) | 1.40 (0.59) | 1.50 (0.60) | 0.655 |
| Daily food intake per person based on Swedish nutrition data adjusted for BMI. Linear regression models adjusted for BMI. Significance level is set to FDR adjusted p-value (q<0.05). Food frequency questionnaire (FFQ). | | | |


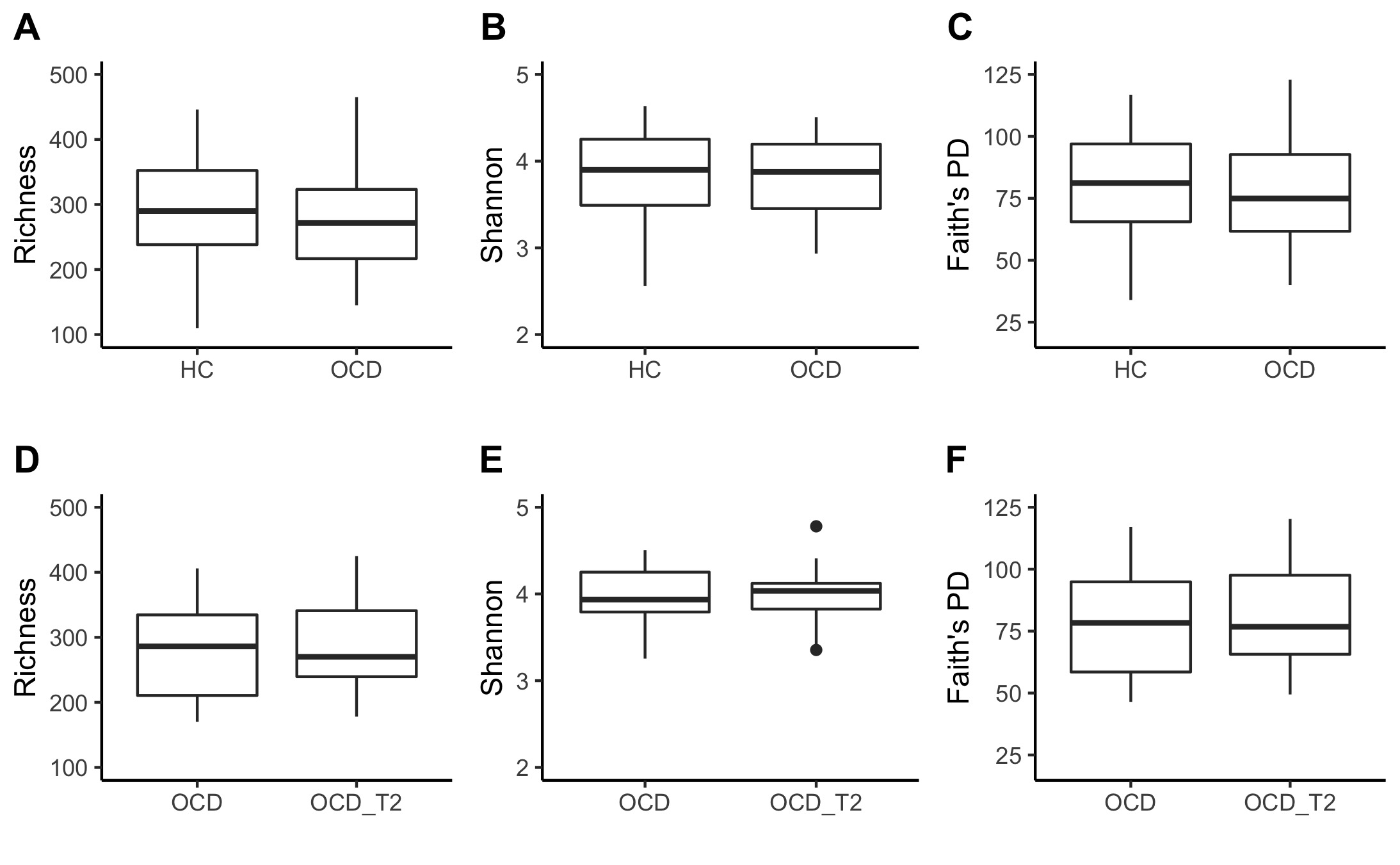
Supplementary Figure 1. Comparison of alpha diversity indices (richness, Shannon index, Faith’s phylogenetic diversity) between healthy controls and patients with OCD (A, B, C), and between patients with OCD before and after CBT treatment (D, E, F).


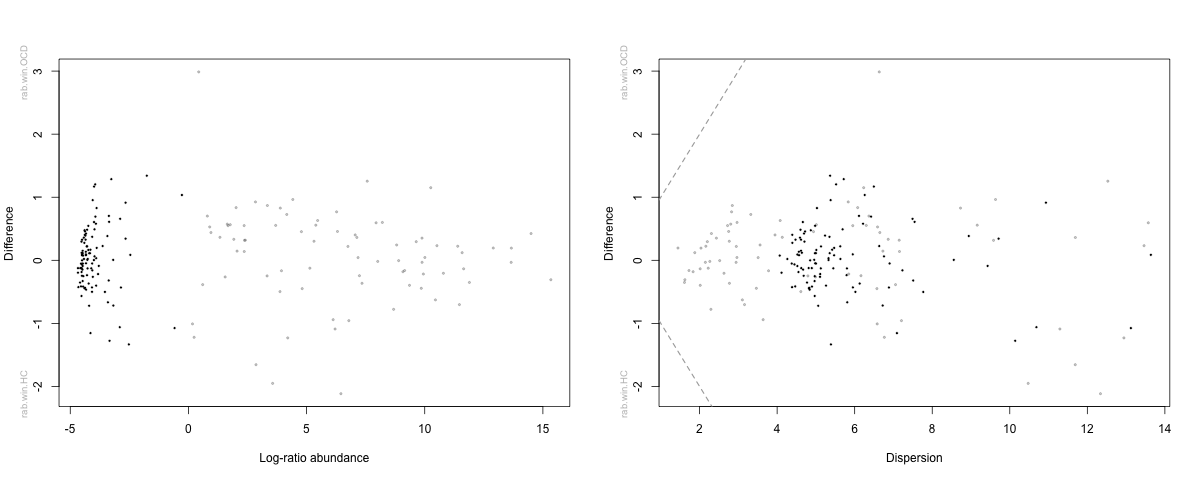


A

B

Supplementary Figure 2. Differences in abundance at the genus level between patients with OCD and healthy controls (HC). (A) The left panel is a Bland-Altman plot that shows the relative abundance of species between OCD before and after ERP. The Log-ratio abundance axis is the center-log-ratio value for the feature. (B) The right panel is an effect plot that shows the difference in effect size (dispersion) between OCD and HC. In both plots, each feature is represented with a dot, grey dots are abundant, while black dots are rare, neither are significantly differentially abundant. Features that are statistically significant are in red (q<0.05).


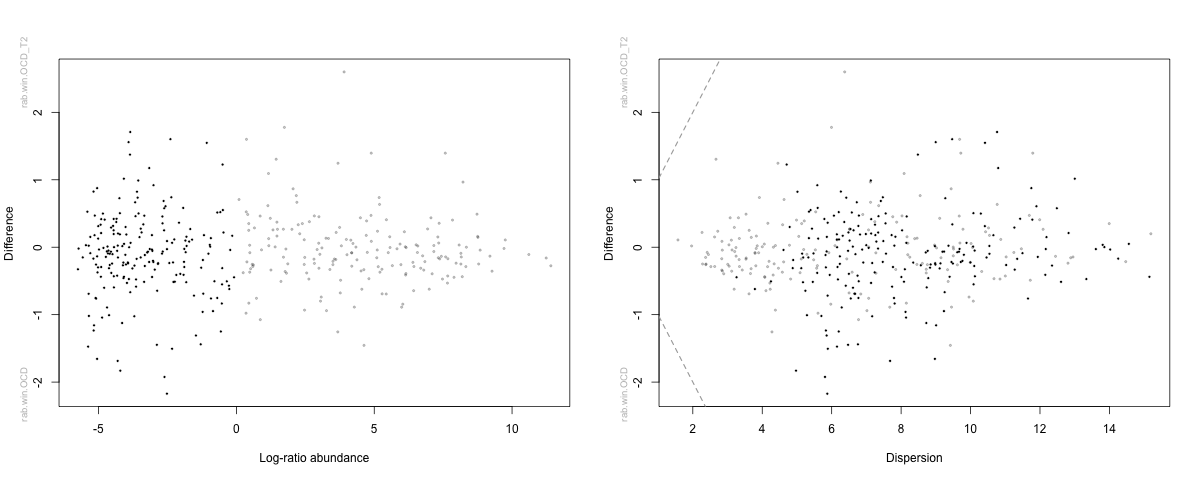


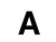

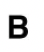


Log-ratio abundance

Dispersion

Difference

Difference

Supplementary Figure 3. Differences in abundance at the species level within patients with OCD before and after ERP. (A) The left panel is a Bland-Altman plot that shows the relative abundance of species between OCD before and after ERP. The Log-ratio abundance axis is the center-log-ratio value for the feature. (B) The right panel is an effect plot that shows the difference in effect size (dispersion) between OCD before and after ERP. In both plots, each feature is represented with a dot, grey dots are abundant, while black dots are rare, neither are significantly differentially abundant. Features that are statistically significant are in red (q<0.05).


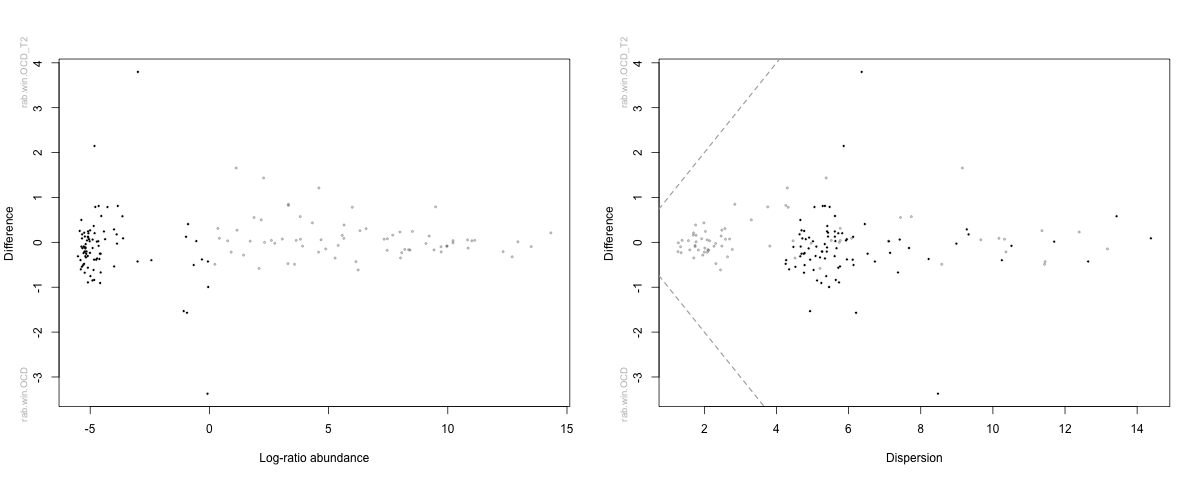


B

A

Supplementary Figure 4. Differences in abundance at the genus level within patients with OCD before and after ERP. (A) The left panel is a Bland-Altman plot that shows the relative abundance of genus between OCD before and after ERP. The Log-ratio abundance axis is the center-log-ratio value for the feature. (B) The right panel is an effect plot that shows the difference in effect size (dispersion) between OCD before and after ERP. In both plots, each feature is represented with a dot, grey dots are abundant, while black dots are rare, neither are significantly differentially abundant. Features that are statistically significant are in red (q<0.05).


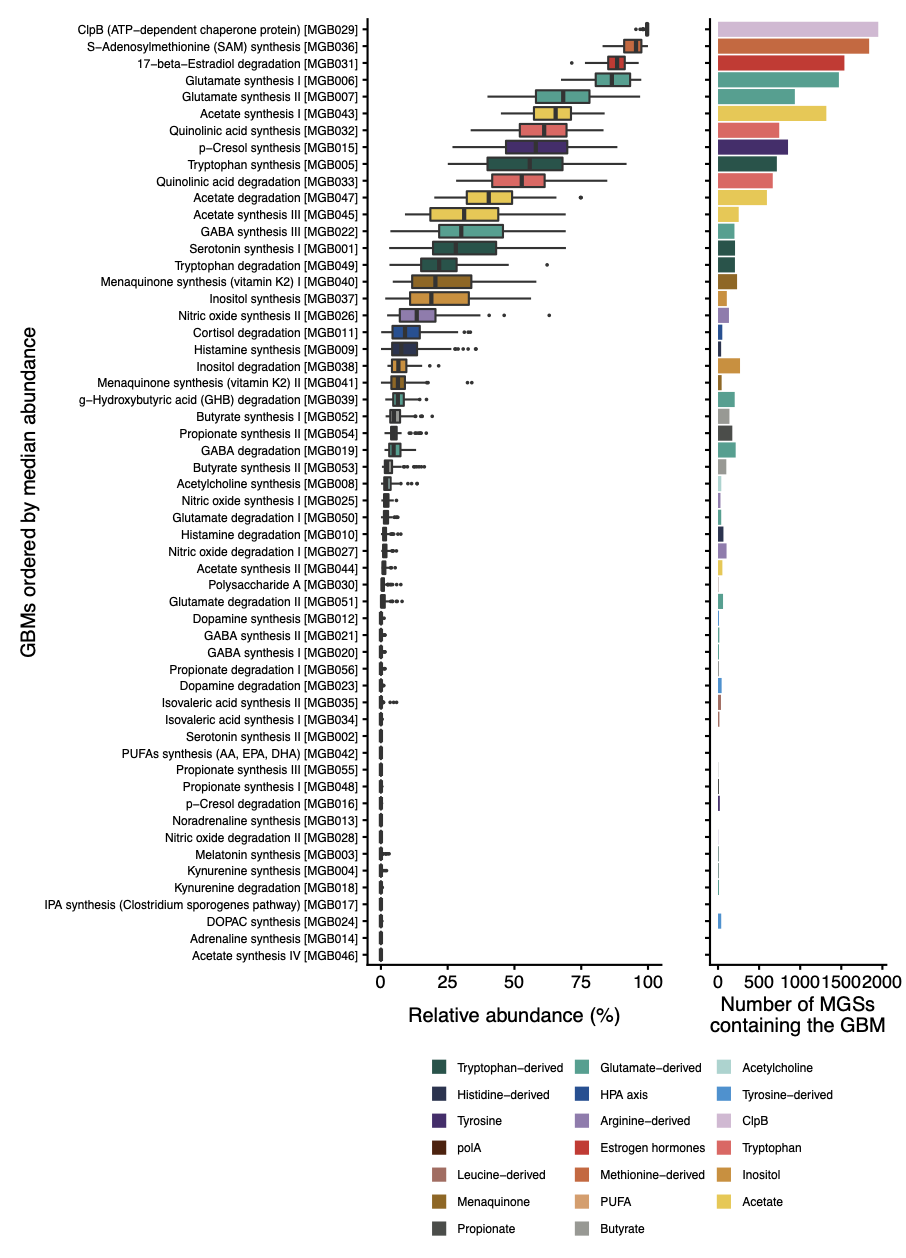


Supplementary Figure 5. Overview of neuroactive potential profiles. The boxplot (left side) shows the relative abundances across samples for each gut-brain module (GBM). The bar plot (right side) depicts the number of MGSs annotated to contain the given GBM. Each GBM is colored by its pathway as described in Valles-Colomer et al. 2019.


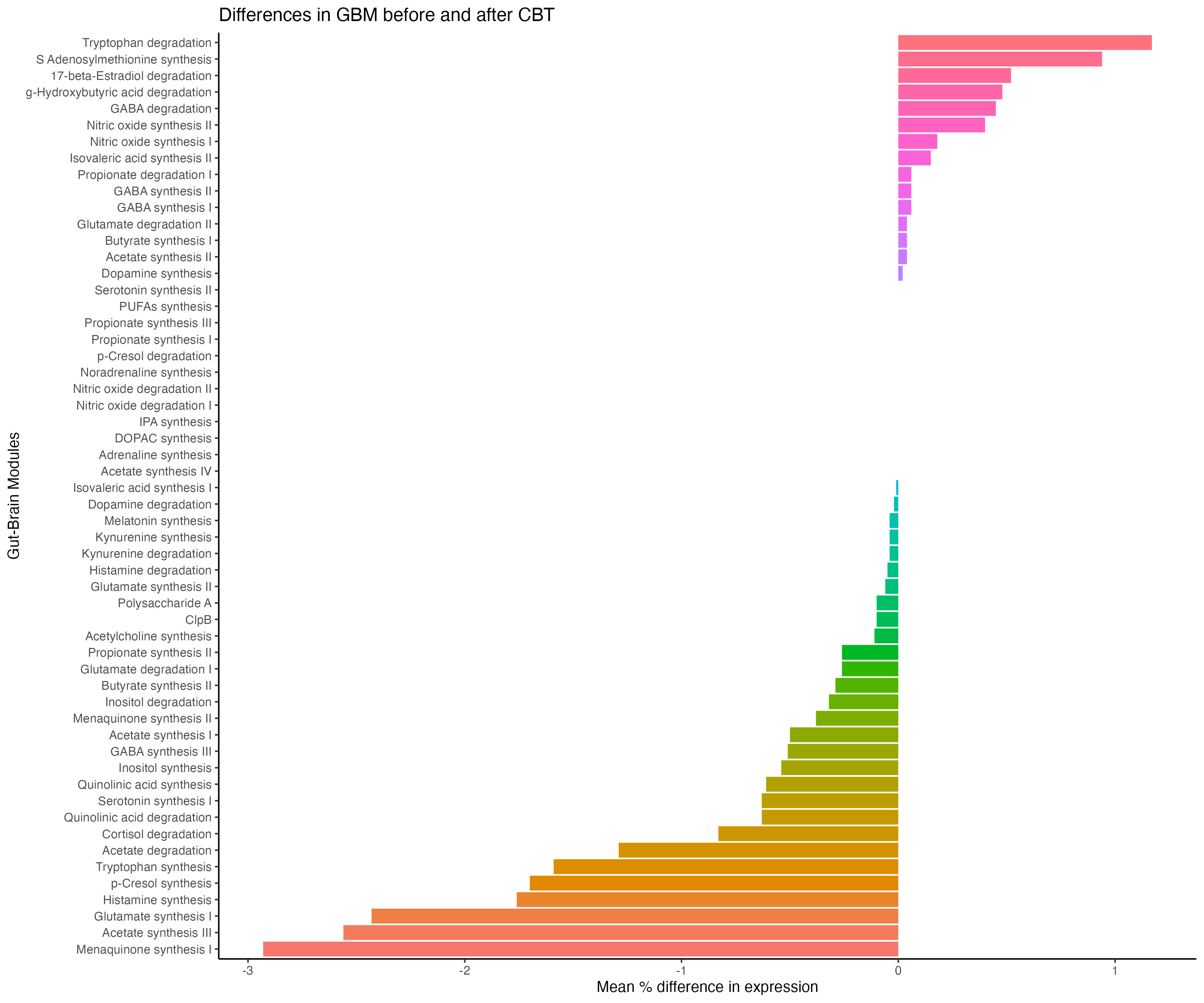


Supplementary Figure 6. Differences in expression of neuroactive gut-brain module (GBM) between patients with OCD at baseline compared with same patients one month after ERP treatment depicted as mean percentage. None of the GBMs reached statistical significance after correcting for multiple comparisons using FDR adjusted p-value (q<0.05).

| Supplementary Table 4. Differences in expression of GBM between patients with OCD and healthy controls | | | | | | |
| --- | --- | --- | --- | --- | --- | --- |
| Gut-brain module (GBM) | estimate | Standard error | Confidence interval low | Confidence  Interval high | q-value | p-value |
| Acetylcholine synthesis | 1.93 | 0.65 | 0.64 | 3.23 | 0.22 | p=.0039 |
| g-Hydroxybutyric acid degradation | -1.5 | 0.81 | -3.12 | 0.12 | 1 | p=.0682 |
| Polysaccharide A | 0.58 | 0.32 | -0.05 | 1.21 | 1 | p=.0718 |
| Nitric oxide synthesis II | 5.1 | 2.82 | -0.51 | 10.71 | 1 | p=.074 |
| Serotonin synthesis I | 7.09 | 3.92 | -0.72 | 14.9 | 1 | p=.0745 |
| GABA synthesis III | 7.11 | 3.95 | -0.75 | 14.97 | 1 | p=.0755 |
| GABA synthesis I | 0.12 | 0.07 | -0.01 | 0.26 | 1 | p=.0784 |
| GABA synthesis II | 0.12 | 0.07 | -0.02 | 0.26 | 1 | p=.0804 |
| Isovaleric acid synthesis I | 0.03 | 0.02 | 0 | 0.06 | 1 | p=.0839 |
| GABA degradation | -1.15 | 0.67 | -2.49 | 0.19 | 1 | p=.0913 |
| Propionate degradation I | 0.12 | 0.07 | -0.02 | 0.25 | 1 | p=.0963 |
| Glutamate degradation II | 0.63 | 0.39 | -0.14 | 1.4 | 1 | p=.1095 |
| Tryptophan degradation | 4.48 | 2.77 | -1.04 | 10.01 | 1 | p=.1104 |
| Kynurenine degradation | 0.02 | 0.01 | -0.01 | 0.05 | 1 | p=.1201 |
| Histamine degradation | 0.5 | 0.33 | -0.15 | 1.15 | 1 | p=.1278 |
| Nitric oxide synthesis I | 0.44 | 0.3 | -0.15 | 1.03 | 1 | p=.1379 |
| Isovaleric acid synthesis II | -0.32 | 0.23 | -0.78 | 0.15 | 1 | p=.1787 |
| Propionate synthesis I | 0.01 | 0.01 | -0.01 | 0.04 | 1 | p=.1837 |
| Dopamine synthesis | -0.05 | 0.04 | -0.13 | 0.03 | 1 | p=.1848 |
| Propionate synthesis III | 0 | 0 | 0 | 0 | 1 | p=.2154 |
| 17-beta-Estradiol degradation | 1.36 | 1.15 | -0.93 | 3.64 | 1 | p=.2414 |
| Propionate synthesis II | 0.95 | 0.83 | -0.7 | 2.6 | 1 | p=.2565 |
| Menaquinone synthesis II | 1.58 | 1.45 | -1.32 | 4.48 | 1 | p=.2803 |
| p-Cresol degradation | 0.01 | 0.01 | -0.01 | 0.02 | 1 | p=.3196 |
| Nitric oxide degradation II | 0 | 0 | 0 | 0 | 1 | p=.3265 |
| Cortisol degradation | 1.88 | 2.17 | -2.44 | 6.2 | 1 | p=.3891 |
| Butyrate synthesis II | 0.68 | 0.91 | -1.13 | 2.49 | 1 | p=.4588 |
| ClpB | 0.14 | 0.19 | -0.24 | 0.51 | 1 | p=.4666 |
| Acetate degradation | 2.17 | 3.06 | -3.92 | 8.27 | 1 | p=.4797 |
| Glutamate synthesis I | 1.24 | 1.95 | -2.64 | 5.12 | 1 | p=.5261 |
| Melatonin synthesis | -0.09 | 0.14 | -0.38 | 0.2 | 1 | p=.5309 |
| Kynurenine synthesis | 0.05 | 0.08 | -0.11 | 0.2 | 1 | p=.55 |
| S-Adenosylmethionine synthesis | -0.67 | 1.16 | -2.98 | 1.65 | 1 | p=.5671 |
| Inositol degradation | -0.56 | 1 | -2.54 | 1.43 | 1 | p=.5781 |
| Butyrate synthesis I | 0.5 | 0.92 | -1.34 | 2.33 | 1 | p=.5915 |
| Inositol synthesis | 1.66 | 3.63 | -5.57 | 8.89 | 1 | p=.648 |
| Quinolinic acid synthesis | -1.4 | 3.25 | -7.87 | 5.07 | 1 | p=.6675 |
| Nitric oxide degradation I | 0.13 | 0.3 | -0.47 | 0.73 | 1 | p=.6684 |
| Acetate synthesis III | 1.6 | 3.84 | -6.05 | 9.25 | 1 | p=.6777 |
| Histamine synthesis | 0.89 | 2.32 | -3.73 | 5.52 | 1 | p=.7015 |
| p-Cresol synthesis | 1.33 | 3.77 | -6.17 | 8.84 | 1 | p=.7243 |
| Acetate synthesis I | -0.82 | 2.43 | -5.66 | 4.01 | 1 | p=.7354 |
| Menaquinone synthesis I | -0.92 | 3.32 | -7.54 | 5.7 | 1 | p=.783 |
| Quinolinic acid degradation | -0.76 | 3.32 | -7.37 | 5.86 | 1 | p=.8207 |
| DOPAC synthesis | 0 | 0.01 | -0.02 | 0.02 | 1 | p=.873 |
| Glutamate synthesis II | 0.41 | 3.3 | -6.16 | 6.98 | 1 | p=.902 |
| Dopamine degradation | 0 | 0.05 | -0.09 | 0.1 | 1 | p=.9497 |
| Glutamate degradation I | 0 | 0.35 | -0.69 | 0.7 | 1 | p=.9898 |
| Acetate synthesis II | 0 | 0.24 | -0.47 | 0.47 | 1 | p=.9957 |
| Tryptophan synthesis | 0.01 | 4.27 | -8.5 | 8.52 | 1 | p=.9981 |
| Serotonin synthesis II | 0 | 0 | 0 | 0 | NA | p=NA |
| Noradrenaline synthesis | 0 | 0 | 0 | 0 | NA | p=NA |
| Adrenaline synthesis | 0 | 0 | 0 | 0 | NA | p=NA |
| IPA synthesis | 0 | 0 | 0 | 0 | NA | p=NA |
| PUFAs synthesis | 0 | 0 | 0 | 0 | NA | p=NA |
| Acetate synthesis IV | 0 | 0 | 0 | 0 | NA | p=NA |
| Estimate equals mean % difference in expression of the gut-brain module (GBM). Significance level is set to FDR adjusted p-value (q<0.05). | | | | | | |

|  | | | | | | |
| --- | --- | --- | --- | --- | --- | --- |
| Supplementary Table 5. Differences in expression of GBM within patients with OCD before and after CBT | | | | | | |
| Gut-brain modules (GBM) | estimate | std.error | conf.low | conf.high | q-value | p-value |
| Propionate synthesis I | 0 | 0 | 0 | 0 | 1 | p=.035 |
| Kynurenine degradation | -0.04 | 0.03 | -0.11 | 0.03 | 1 | p=.278 |
| Glutamate synthesis I | -2.43 | 2.57 | -7.68 | 2.83 | 1 | p=.353 |
| Dopamine degradation | -0.02 | 0.03 | -0.09 | 0.04 | 1 | p=.452 |
| Propionate synthesis III | 0 | 0 | 0 | 0 | 1 | p=.486 |
| Isovaleric acid synthesis II | 0.15 | 0.24 | -0.34 | 0.64 | 1 | p=.54 |
| Menaquinone synthesis I | -2.93 | 4.88 | -12.93 | 7.07 | 1 | p=.553 |
| Histamine synthesis | -1.76 | 2.98 | -7.86 | 4.34 | 1 | p=.559 |
| Polysaccharide A | -0.1 | 0.18 | -0.46 | 0.26 | 1 | p=.577 |
| S-Adenosylmethionine synthesis | 0.94 | 1.76 | -2.67 | 4.54 | 1 | p=.599 |
| DOPAC synthesis | 0 | 0.01 | -0.01 | 0.01 | 1 | p=.606 |
| ClpB | -0.1 | 0.19 | -0.5 | 0.3 | 1 | p=.613 |
| GABA synthesis I | 0.06 | 0.11 | -0.17 | 0.29 | 1 | p=.613 |
| p-Cresol degradation | 0 | 0 | 0 | 0 | 1 | p=.614 |
| GABA synthesis II | 0.06 | 0.11 | -0.17 | 0.29 | 1 | p=.616 |
| Propionate degradation I | 0.06 | 0.11 | -0.18 | 0.29 | 1 | p=.622 |
| Dopamine synthesis | 0.02 | 0.04 | -0.06 | 0.09 | 1 | p=.623 |
| Glutamate degradation I | -0.26 | 0.54 | -1.36 | 0.84 | 1 | p=.636 |
| Acetate synthesis III | -2.56 | 5.55 | -13.93 | 8.81 | 1 | p=.648 |
| Nitric oxide synthesis I | 0.18 | 0.41 | -0.66 | 1.03 | 1 | p=.661 |
| g-Hydroxybutyric acid degradation | 0.48 | 1.11 | -1.8 | 2.76 | 1 | p=.668 |
| GABA degradation | 0.45 | 1.09 | -1.78 | 2.67 | 1 | p=.683 |
| Isovaleric acid synthesis I | -0.01 | 0.03 | -0.06 | 0.04 | 1 | p=.703 |
| 17-beta-Estradiol degradation | 0.52 | 1.45 | -2.44 | 3.49 | 1 | p=.721 |
| Acetate degradation | -1.29 | 3.72 | -8.91 | 6.33 | 1 | p=.732 |
| Tryptophan degradation | 1.17 | 3.8 | -6.6 | 8.95 | 1 | p=.76 |
| Cortisol degradation | -0.83 | 2.69 | -6.35 | 4.69 | 1 | p=.76 |
| p-Cresol synthesis | -1.7 | 5.75 | -13.48 | 10.08 | 1 | p=.77 |
| Inositol degradation | -0.32 | 1.14 | -2.66 | 2.03 | 1 | p=.784 |
| Tryptophan synthesis | -1.59 | 6.03 | -13.95 | 10.77 | 1 | p=.794 |
| Histamine degradation | -0.05 | 0.24 | -0.54 | 0.44 | 1 | p=.829 |
| Kynurenine synthesis | -0.04 | 0.17 | -0.39 | 0.32 | 1 | p=.832 |
| Acetylcholine synthesis | -0.11 | 0.56 | -1.27 | 1.05 | 1 | p=.847 |
| Propionate synthesis II | -0.26 | 1.41 | -3.15 | 2.63 | 1 | p=.854 |
| Butyrate synthesis II | -0.29 | 1.62 | -3.62 | 3.03 | 1 | p=.858 |
| Melatonin synthesis | -0.04 | 0.27 | -0.6 | 0.51 | 1 | p=.877 |
| Acetate synthesis I | -0.5 | 3.56 | -7.78 | 6.79 | 1 | p=.89 |
| Serotonin synthesis I | -0.63 | 4.69 | -10.23 | 8.97 | 1 | p=.894 |
| Menaquinone synthesis II | -0.38 | 2.84 | -6.19 | 5.43 | 1 | p=.894 |
| Quinolinic acid degradation | -0.63 | 4.71 | -10.28 | 9.02 | 1 | p=.895 |
| Quinolinic acid synthesis | -0.61 | 4.89 | -10.63 | 9.41 | 1 | p=.902 |
| Glutamate degradation II | 0.04 | 0.34 | -0.65 | 0.73 | 1 | p=.912 |
| GABA synthesis III | -0.51 | 4.69 | -10.12 | 9.09 | 1 | p=.914 |
| Acetate synthesis II | 0.04 | 0.42 | -0.81 | 0.9 | 1 | p=.918 |
| Nitric oxide synthesis II | 0.4 | 3.94 | -7.67 | 8.47 | 1 | p=.92 |
| Inositol synthesis | -0.54 | 5.47 | -11.74 | 10.66 | 1 | p=.922 |
| Butyrate synthesis I | 0.04 | 1.55 | -3.15 | 3.22 | 1 | p=.982 |
| Glutamate synthesis II | -0.06 | 4.75 | -9.79 | 9.67 | 1 | p=.99 |
| Nitric oxide degradation I | 0 | 0.55 | -1.12 | 1.13 | 1 | p=.997 |
| Serotonin synthesis II | 0 | 0 | 0 | 0 | NA | p=NA |
| Noradrenaline synthesis | 0 | 0 | 0 | 0 | NA | p=NA |
| Adrenaline synthesis | 0 | 0 | 0 | 0 | NA | p=NA |
| IPA synthesis | 0 | 0 | 0 | 0 | NA | p=NA |
| Nitric oxide degradation II | 0 | 0 | 0 | 0 | NA | p=NA |
| PUFAs synthesis | 0 | 0 | 0 | 0 | NA | p=NA |
| Acetate synthesis IV | 0 | 0 | 0 | 0 | NA | p=NA |
| Estimate equals mean % difference in expression of the gut-brain module (GBM). Significance level is set to FDR adjusted p-value (q<0.05). | | | | | | |
